# Quantitative CT Analysis of Nasal Septal Body and Inferior Turbinate Morphology in Allergic Rhinitis: Structural–Airway Relationships

**DOI:** 10.3390/diagnostics16213547

**Published:** 2026-10-28

**Authors:** İbrahim Hıra, Hilal Melis Altıntaş

**Affiliations:** 1 Private Otorhinolaryngology Clinic, 06510 Ankara, Türkiye; dr.ibrahimhira@gmail.com; 2 Department of Audiology, Faculty of Health Sciences, Ankara Medipol University, 06050 Ankara, Türkiye; 3 Department of Anatomy, Faculty of Medicine, Ankara Medipol University, 06050 Ankara, Türkiye

**Keywords:** allergic rhinitis, nasal septal body, inferior turbinate, computed tomography, nasal obstruction, morphometry

## Abstract

**Background/Objectives:** Allergic rhinitis (AR) is characterized by dynamic mucosal congestion, yet the nasal septal body (NSB), inferior turbinate (IT), and adjacent airway passages have rarely been assessed together. We evaluated whether AR is associated with a multilevel structural–airway pattern while limiting confounding by marked septal deviation. **Methods:** This retrospective cross-sectional study included 301 adults with septal deviation < 8° (163 patients with clinically diagnosed AR and 138 participants in a clinically defined non-AR comparison group). Coronal computed tomography measurements included total transverse NSB width, dominant IT width, and adjacent airway widths. Between-group differences were evaluated using effect sizes and multivariable regression adjusted for age, sex, and septal deviation angle. Correlation, interaction, and sensitivity analyses assessed structure–airway relationships. **Results:** Patients with AR had greater NSB width (11.92 ± 1.81 vs. 10.80 ± 1.89 mm; Hedges’ *g* = 0.605) and dominant IT width (12.78 ± 1.69 vs. 10.41 ± 1.83 mm; *g* = 1.350), together with smaller adjacent airway widths. All four primary comparisons remained significant after Holm correction and adjustment (all *p* < 0.001). The NSB–IT correlation (rho = 0.257) was attenuated after adjustment for AR (partial rho = 0.089; q = 0.124), whereas inverse structure–airway associations persisted. A significant AR-status × IT-width interaction was observed for medial airway width (interaction q = 0.030). **Conclusions:** At the time of CT, patients with AR had greater NSB and IT widths and smaller corresponding regional airway dimensions, with a larger between-group morphometric difference at the IT level. These findings support a multilevel anatomical view of the nasal airway in AR, in which the NSB and IT are best interpreted together with their adjacent airway spaces rather than as isolated structures. Their clinical relevance, however, requires validation against symptoms and objective airflow measures.

## 1. Introduction

Allergic rhinitis (AR) is a highly prevalent IgE-mediated inflammatory disorder of the nasal mucosa in which nasal obstruction, rhinorrhea, sneezing, and itching may substantially impair sleep, daily functioning, and quality of life [1,2,3,4]. Nasal obstruction in AR is not simply a consequence of a fixed reduction in airway caliber. Allergen-driven epithelial barrier disruption and immune activation are central to AR pathophysiology [5,6]. Mast-cell mediators promote vasodilatation, increased vascular permeability, tissue edema, and glandular secretion, contributing to dynamic changes in mucosal volume [6]. These processes may alter the geometry of the nasal passages over short and longer time scales and may coexist with pre-existing structural factors. Accordingly, contemporary assessment of nasal obstruction increasingly recognizes an interaction between inflammatory mucosal disease and anatomy rather than treating them as mutually exclusive causes [1,4].

The inferior turbinate (IT) is the mucosal structure most commonly associated with AR-related congestion because its submucosa contains a rich venous and cavernous vascular network capable of marked volume change. Allergen-specific IgE can be detected within inferior turbinate tissue, demonstrating local allergic sensitization at the turbinate level [7]. Nevertheless, the cross-sectional area available for airflow at any point in the nasal cavity is determined by the relationship between opposing surfaces. Focusing only on the lateral wall or IT may therefore incompletely characterize a patient in whom a medial septal mucosal structure also occupies part of the same functional airway.

The nasal septal body (NSB), also termed the septal swell body (SSB) or septal turbinate, is a localized thickening of the anterior septal mucosa that lies near the internal nasal valve. Histologically, the region contains glandular tissue and venous sinusoids and has been proposed to participate in vasoactive airflow regulation [8]. Cross-sectional imaging studies using MRI and CT have established that the NSB can be identified and quantitatively measured [9,10,11]. Its location within the narrow anterior nasal airway has generated increasing interest in its potential contribution to nasal resistance [8,9,10]. The terminology and exact anatomical interpretation of this structure remain debated: a recent anatomical review argued that the region corresponds to the classical septal tubercle rather than a unique “swell body” organ [12], and a literature review concluded that inconsistent anatomical and histological findings have so far prevented consensus on the clinical significance and management of the NSB [13]. Regardless of nomenclature, the clinically relevant issue is that a reproducibly identifiable septal soft-tissue prominence may influence the available airway adjacent to the internal nasal valve.

Interpretation of NSB and IT dimensions on imaging requires careful attention to septal geometry. Mendoza et al. demonstrated an association between NSB morphology and septal deviation and classified deviation angles of ≤8° as mild using the crista galli–premaxillary angular measurement method [10]. More recent CT and cone-beam CT investigations have similarly shown that septal deviation is associated with side-to-side differences in IT mucosal thickness, bony dimensions, and turbinate volume [14,15,16]. These observations are particularly important when AR is being studied because septal-deviation-related asymmetry may confound observed differences in mucosal dimensions between AR and non-AR groups. A study design that minimizes marked septal deformity and adjusts for residual deviation is therefore preferable when the objective is to reduce anatomical confounding in the estimation of AR-associated morphometric differences.

Previous imaging studies provide evidence that sinonasal inflammatory disease and AR may be associated with differences in both the NSB and IT, but the literature is not uniform. Gelera et al. reported larger NSB dimensions in patients with sinonasal disease than in controls [11]. Hizli et al. found greater NSB area and width in patients with IT hypertrophy and greater NSB measurements in those with concomitant AR, while Çoban et al. also reported larger NSB dimensions in AR and other sinonasal pathologies [17,18]. By contrast, a smaller CT series found no difference in NSB width between 40 patients with AR and 30 controls [19], and one radiological comparison of allergic and non-allergic rhinitis did not demonstrate greater IT dimensions in allergic disease [20]. More recently, Hirai et al. measured the NSB, the IT, and the air column adjacent to each on CT in 152 surgical patients and showed that all four widths vary with the side and degree of septal deviation; AR was recorded only as a comorbidity [21]. Histological data nevertheless support allergic mucosal differences in the IT, including edema and inflammatory-cell patterns consistent with remodeling [22]. Thus, the existing literature indicates that each structure can differ across disease groups, but does not fully resolve whether the NSB and IT participate in a coordinated AR-associated structural pattern or how their dimensions relate to the immediately adjacent airway at their respective levels. Moreover, these studies differed in how AR was defined and how septal deviation was handled, only one of them measured the NSB together with the air column adjacent to it, and that study addressed septal deviation rather than AR; the reproducibility of the NSB measurement was not formally reported in any of them.

The physiological nasal cycle further complicates interpretation of static imaging. Serial CT observations have shown that changes in NSB width can occur in parallel with changes in IT size within the same individual, suggesting that both structures participate to some extent in dynamic nasal mucosal behavior [23]. This finding makes simultaneous assessment particularly relevant: a correlation between NSB and IT dimensions in a cross-sectional cohort may reflect underlying anatomical covariation, shared associations with AR status, the phase of the nasal cycle, or a combination of these factors. Evaluating the relationship before and after adjustment for AR can therefore provide information about the extent to which an apparent NSB–IT association contains a between-group component.

CT offers high-resolution anatomical information but should not be interpreted as a direct measure of nasal airflow or symptom burden. Assessment of nasal obstruction may include objective tests such as rhinomanometry, acoustic rhinometry, and peak nasal inspiratory flow, together with validated patient-reported outcome measures; these approaches assess different aspects of nasal patency and obstruction [24,25,26]. A recent *Diagnostics* study comparing acoustic rhinometry with CT found moderate correspondence between cross-sectional measurements but also systematic differences, with acoustic rhinometry generally underestimating CT-derived nasal area, indicating that the two geometric methods are related but not interchangeable [27]. A systematic review similarly found that relationships between clinical examination findings and objective nasal patency measurements vary according to the site and severity of structural obstruction [28]. Within these limits, standardized CT morphometry remains valuable for quantifying structural relationships, particularly when acquisition, reorientation, landmarks, and measurement reproducibility are rigorously controlled. Recent CT-based sinonasal skull-base morphometry published in *Diagnostics* has likewise emphasized multidimensional measurement and interobserver reliability when interpreting complex anatomy [29].

On this background, the present study was designed to evaluate the NSB and IT together with their immediately adjacent airway passages in adults with clinically diagnosed AR and a clinically defined non-AR comparison group while minimizing confounding by marked septal deviation. The primary objective was to determine whether AR is associated with a multilevel structural–airway pattern characterized by concurrent differences in NSB width, dominant IT width, and regional airway dimensions. Secondary objectives were to examine the relationships between the two mucosal structures, quantify the association of each structure with its adjacent airway, and test whether the slopes of these structure–airway relationships differ by AR status. We hypothesized that patients with AR would have greater NSB and IT widths and smaller corresponding airway dimensions, with a larger between-group morphometric difference at the IT level.

## 2. Materials and Methods

### 2.1. Study Design and Population

This study was designed as a retrospective, observational, cross-sectional radiological investigation. The study protocol was approved by the Ankara Medipol University Non-Interventional Clinical Research Ethics Committee (decision no. 153; 16 June 2026), and the study was conducted in accordance with the principles of the Declaration of Helsinki. The requirement for written informed consent was waived by the ethics committee because of the retrospective design and the use of anonymized archived CT images.

The study population comprised adults whose paranasal sinus computed tomography examinations acquired between 2021 and 2026 were retrospectively reviewed. All participants in both groups were identified among patients evaluated in the Department of Otorhinolaryngology.

Participants were classified into a clinically diagnosed AR group and a clinically defined non-AR comparison group (hereafter, the non-AR group). AR was defined from routine clinical records based on a compatible history and nasal symptoms together with findings from nasal examination and/or endoscopy; skin-prick testing or serum-specific IgE testing was incorporated when it had been performed for clinical indications. Because objective allergy testing was not applied uniformly in this retrospective cohort, the AR classification reflects routine clinical diagnosis rather than a protocol-mandated sensitization test. The exact numbers of patients who underwent skin-prick testing or serum-specific IgE testing could not be determined from the available retrospective records. The clinically defined non-AR comparison group comprised individuals with no documented history of allergic rhinitis, no clinical evidence of active sinonasal inflammatory disease, and paranasal sinus CT images of adequate diagnostic quality. Group membership was based on clinical records rather than systematic allergy testing; therefore, the absence of atopy was not objectively confirmed. According to the department’s standard practice, paranasal sinus CT examinations were performed before medical treatment for rhinitis was initiated. Under this practice, CT was not performed in patients receiving active treatment for rhinitis, because treatment could influence the dimensions of the structures evaluated; consequently, patients under active treatment at the time of CT did not enter the study population. However, medication use was not recorded as a study variable, and the interval between discontinuation of any previous rhinitis medication and imaging could not be verified retrospectively; therefore, residual medication effects cannot be excluded.

### 2.2. Inclusion and Exclusion Criteria

Individuals aged 18 years or older who had paranasal sinus computed tomography scans of adequate image quality and accessible clinical data were included in the study. The septal deviation angle was measured objectively in all participants. To minimize confounding related to septal geometry, only patients with a septal deviation angle < 8° were included. This conservative threshold was selected to restrict the cohort to the mild-deviation range; Mendoza et al. classified deviation angles of ≤8° as mild using the same crista galli–premaxillary angular measurement approach [10]. Patients with a septal deviation angle ≥ 8°, a septal spur, or marked septal asymmetry were excluded.

Patients with a history of septoplasty, rhinoplasty, inferior turbinate surgery, or endoscopic sinus surgery were also excluded, as were those with sinonasal polyps, chronic rhinosinusitis, an acute upper respiratory tract infection, a nasal mass, maxillofacial trauma, a craniofacial anomaly, or imaging artifacts.

### 2.3. Radiological Assessment

All CT examinations were acquired on the same scanner (Alexion; Toshiba Medical Systems, Otawara, Japan) using an unchanged helical acquisition and reconstruction protocol throughout the 2021–2026 study period. The acquisition parameters were 120 kVp, 100 mA, a 0.75-s rotation time (75 mAs), a pitch factor of 0.688, and a volume CT dose index of 29.4 mGy, with a display field of view of 177.5 mm and a 512 × 512 reconstruction matrix corresponding to an in-plane pixel size of 0.346 × 0.346 mm. Axial images were reconstructed at a 0.5-mm section thickness with a bone algorithm (FC30), and coronal multiplanar reconstructions with a 2.0-mm section thickness were used for morphometric assessment. All measurements were performed in a bone window (window width 2700 HU, window level 350 HU). Image reorientation and all morphometric measurements were performed using 3D Slicer (version 5.12.3; https://download.slicer.org/; Brigham and Women’s Hospital, Boston, MA, USA).

Before measurement, each CT dataset was reoriented using multiplanar reconstruction to reduce variability caused by head rotation, lateral tilt, and flexion or extension. In the sagittal view, the axial reference plane was aligned parallel to the Frankfort horizontal plane, defined by the inferior orbital rim and the superior margin of the external auditory canal. In the coronal view, the sagittal reference plane was aligned with the midsagittal plane passing through the crista galli and anterior nasal spine. The coronal reference plane was then positioned perpendicular to both the axial and sagittal reference planes.

Before formal measurements, the two observers held a consensus session to standardize the anatomical landmarks and measurement definitions. All primary measurements were performed by an anatomist with 10 years of experience who was blinded to the patients’ clinical group assignments. For interobserver reliability, a radiologist with 25 years of experience independently remeasured a randomly selected subset of 50 examinations while blinded to the clinical group and the first observer’s measurements.

The septal deviation angle was measured on coronal computed tomography images as the angle between the crista galli–premaxilla line and the line extending from the crista galli to the most lateralized point of the nasal septum. Cases meeting the predefined septal deviation exclusion criterion or presenting with a septal spur were excluded to minimize the potential influence of septal geometry on NSB and IT morphology.

**Figure 1:**
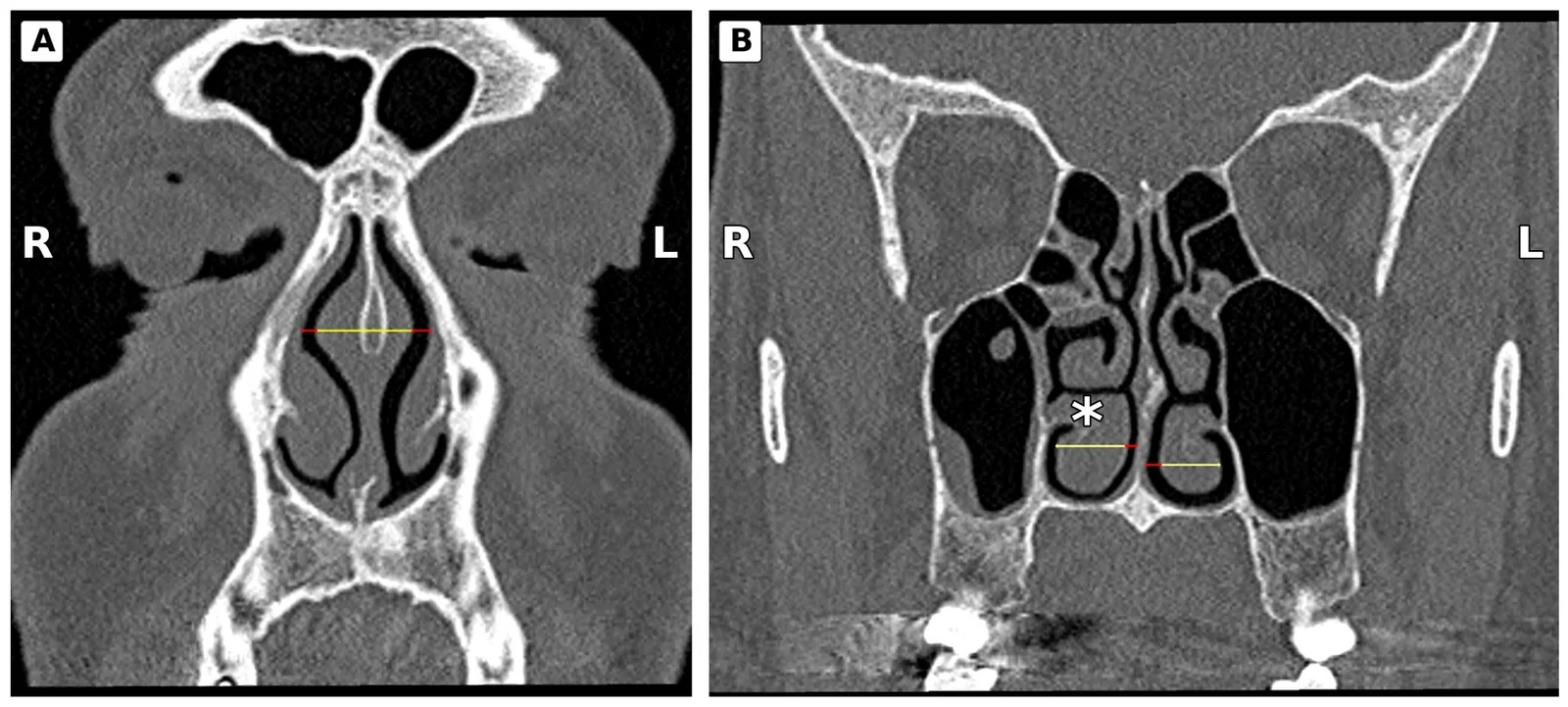
Illustration of the morphometric measurements on coronal paranasal sinus computed tomography images. (**A**) The yellow line indicates the total transverse width of the nasal septal body (NSB) at its widest level, and the red lines indicate the bilateral airway passages lateral to the NSB at the same level. (**B**) The yellow lines indicate the widths of the inferior turbinates (ITs) at their widest points, and the red lines indicate the airway passages between the ITs and the nasal septum at the same level. The asterisk marks the dominant (wider) IT, which was used for the primary patient-level analysis. R and L denote the patient’s right and left sides (radiological convention). NSB, nasal septal body; IT, inferior turbinate.

For evaluation of the nasal septal body (NSB), the coronal section on which the NSB appeared most prominent and widest was selected (Figure 1A). A single measurement, termed the total transverse NSB width (hereafter, NSB width), was recorded on this section and was defined as the greatest horizontal distance between the right and left lateral mucosal surfaces of the NSB, measured perpendicular to the midsagittal plane (Figure 1A). Because the NSB lies over the anterior septum, where the septal skeleton is cartilaginous rather than bony, and because the cartilaginous septum cannot be reliably discriminated from the overlying mucosa on CT, the mucosal and cartilaginous components could not be separated; the measurement was therefore recorded as a single transverse dimension comprising the mucosa of both sides together with the intervening cartilaginous septum. Separate right-sided and left-sided NSB components were not measured.

Upper nasal airway width was assessed on the same coronal section and at the same horizontal level used for NSB measurement. It was defined as the width of the air column between the lateral border of the NSB and the lateral nasal wall (Figure 1A). The right and left airway widths were recorded separately, and their arithmetic mean was calculated as the patient-level mean airway width adjacent to the NSB.

For evaluation of the inferior turbinates, the maximum right and left IT widths were first compared, and the coronal section on which the numerically wider (dominant) IT appeared widest was selected (Figure 1B). On this section, the width of the dominant IT and that of the contralateral IT were measured and were defined as the greatest horizontal distance between the medial and lateral mucosal surfaces of each turbinate. The dominant side used in the primary patient-level analysis was therefore assigned from the numerical comparison of right and left IT widths, irrespective of the side designation initially recorded by the evaluator. This strategy was chosen to represent the side with the greatest local turbinate burden; bilateral analyses were retained as sensitivity analyses to determine whether the findings depended on this side-selection approach.

Medial airway width was measured on the same coronal section used for IT assessment and was defined as the width of the air column between the nasal septum and the medial surface of the IT (Figure 1B). Right and left medial airway widths were recorded separately. For the primary patient-level analysis, the medial airway width ipsilateral to the dominant IT was used so that turbinate size and the corresponding local air column were evaluated on the same side. Bilateral measurements were retained for sensitivity analyses.

Figure 1 summarizes the standardized measurement framework. At the NSB level, the structure and the immediately adjacent bilateral air columns were quantified on the same reoriented coronal image; at the IT level, turbinate width and the corresponding medial airway were measured on the same coronal section. This paired structure–airway approach preserved local anatomical correspondence between each mucosal structure and the regional air column.

### 2.4. Assessment of Measurement Reliability

For intraobserver reliability, the anatomist repeated the measurements in the same subset of 50 patients after a 3-week interval without access to the initial measurements. For continuous variables, interobserver reliability was evaluated using a two-way random-effects model for single-measure absolute agreement, whereas intraobserver reliability was assessed using a two-way mixed-effects model for single-measure absolute agreement. Intraclass correlation coefficients (ICCs) with 95% confidence intervals were calculated. Agreement for dominant IT side classification was evaluated using percentage agreement and Cohen’s kappa. Absolute agreement was further characterized using the mean paired difference (bias), 95% limits of agreement, and the 95% minimum detectable change (MDC95). For intraobserver analyses, paired differences were calculated between the repeated and initial measurements by the same observer; for interobserver analyses, differences were calculated between the second observer and the primary observer. The 95% limits of agreement were calculated as the mean bias ± 1.96 times the standard deviation of the paired differences. The standard error of measurement (SEM) was calculated as SD × √(1 − ICC), and MDC95 was calculated as 1.96 × √2 × SEM. Bootstrap 95% confidence intervals for the ICCs, the mean bias, and Cohen’s kappa were obtained from 5000 patient-level resamples. These analyses were performed separately for each continuous morphometric variable.

### 2.5. Statistical Analysis

The distributions of continuous variables were assessed within each study group using histograms, Q–Q plots, and the Shapiro–Wilk test. Approximately symmetric width measurements were summarized as mean ± standard deviation and compared using Welch’s independent-samples *t* test. Right-skewed airway measurements were summarized as median and interquartile range and compared using the Mann–Whitney *U* test. Mean differences with 95% confidence intervals and Hedges’ *g* were reported for width measurements, whereas Hodges–Lehmann location shifts with bootstrap 95% confidence intervals (2000 resamples) and rank-biserial correlation coefficients were reported for airway measurements. The four prespecified primary outcomes were total transverse NSB width, mean airway width adjacent to the NSB, dominant IT width, and medial airway width ipsilateral to the dominant IT. Holm correction was applied to the four primary between-group comparisons.

Independent associations between AR and the primary outcomes were evaluated using multiple linear regression models adjusted for age, sex, and septal deviation angle. HC3 heteroscedasticity-robust standard errors were used, and adjusted effects were reported as mean differences between the AR and non-AR groups in millimeters. Associations between anatomical and airway measurements were examined using Spearman correlations. Partial Spearman correlations were adjusted first for age, sex, and septal deviation angle and then additionally for AR status. The Benjamini–Hochberg false discovery rate procedure was applied within the prespecified families of correlation analyses.

Interactions with AR status were evaluated using regression models containing the relevant AR-status × measurement interaction term. In sensitivity analyses incorporating bilateral measurements, standard errors were clustered at the patient level and side was included as a fixed effect. Airway measurements of 0 mm, which represented true mucosal contact between opposing surfaces rather than missing values, were retained as valid observations in the primary analyses; the adjusted airway comparisons were then repeated after excluding the patients with such a measurement. Model assumptions and influential observations were assessed using residual plots and Cook’s distance.

Because this was a retrospective study including all eligible cases, no a priori sample size calculation or post hoc power analysis based on observed effect sizes was performed. A sensitivity analysis based on the noncentral t distribution indicated that, with a two-sided α of 0.05 and 80% power, the available group sizes were sufficient to detect a standardized between-group difference of d = 0.325 and a correlation of |r| = 0.160 in the full sample. All tests were two-sided, and *p* < 0.05 was considered statistically significant. Statistical analyses were performed in Python 3.12.13 (pandas 2.2.3, NumPy 2.3.5, and SciPy 1.17.0), and the figures were generated with Matplotlib 3.10.8; a fixed random seed (20260803) was used for all resampling procedures. The dataset was complete for all prespecified variables, and no imputation was required.

### 2.6. Use of Generative Artificial Intelligence

OpenAI ChatGPT (GPT-5.6 Sol; OpenAI, San Francisco, CA, USA) was used to assist with English language proofreading, article formatting, and the writing of the plotting code used to generate the figures from statistical results that had been produced and verified by the authors. ChatGPT was not used for study design, data collection, statistical analysis, or interpretation of the results. All figures were reviewed and verified by the authors against the underlying data and statistical results. The authors have reviewed and edited all AI-assisted output and take full responsibility for the content of the publication.

## 3. Results

### 3.1. Patient and Baseline Characteristics

Of 367 paranasal sinus CT examinations screened, 66 were excluded: 23 because the septal geometry did not meet the predefined criteria (a septal deviation angle ≥8°, a septal spur, or marked septal asymmetry), 12 because of previous septoplasty, inferior turbinate surgery, or endoscopic sinus surgery, 11 because of image artifacts, 7 because of chronic rhinosinusitis, 7 because of age below 18 years, and 6 because of maxillofacial trauma. No examinations were excluded on the remaining criteria. A total of 301 patients were therefore included in the analysis, comprising 163 patients in the AR group and 138 in the non-AR group. No significant differences were observed between the groups in terms of age, sex distribution, or septal deviation angle; the observed septal deviation angles ranged from 1.0° to 6.9°. The distribution of the dominant inferior turbinate side, derived from the numerical width measurements, differed between the groups; this was a secondary comparison that was not adjusted for multiplicity. No missing or negative values were identified in the variables included in the analysis (Table 1).

### 3.2. Measurement Reliability

Measurement reproducibility was high, with intraobserver ICCs ranging from 0.920 to 0.960 and interobserver ICCs ranging from 0.890 to 0.940. The lower bounds of all 95% confidence intervals exceeded 0.75. Agreement for dominant IT side classification was 96% for intraobserver assessment (κ = 0.918) and 92% for interobserver assessment (κ = 0.838). Detailed reliability estimates are provided in Supplementary Table S1.

Supplementary Table S1 provides parameter-specific ICCs together with mean bias, 95% limits of agreement, and the 95% minimum detectable change (MDC95). Across the continuous measurements, intraobserver and interobserver mean biases were small, and the bootstrap 95% confidence interval for the bias included zero for every parameter, providing no evidence of systematic measurement shift and complementing the high ICC estimates with an assessment of absolute measurement agreement.

### 3.3. Primary Morphometric Outcomes

**Figure 2:**
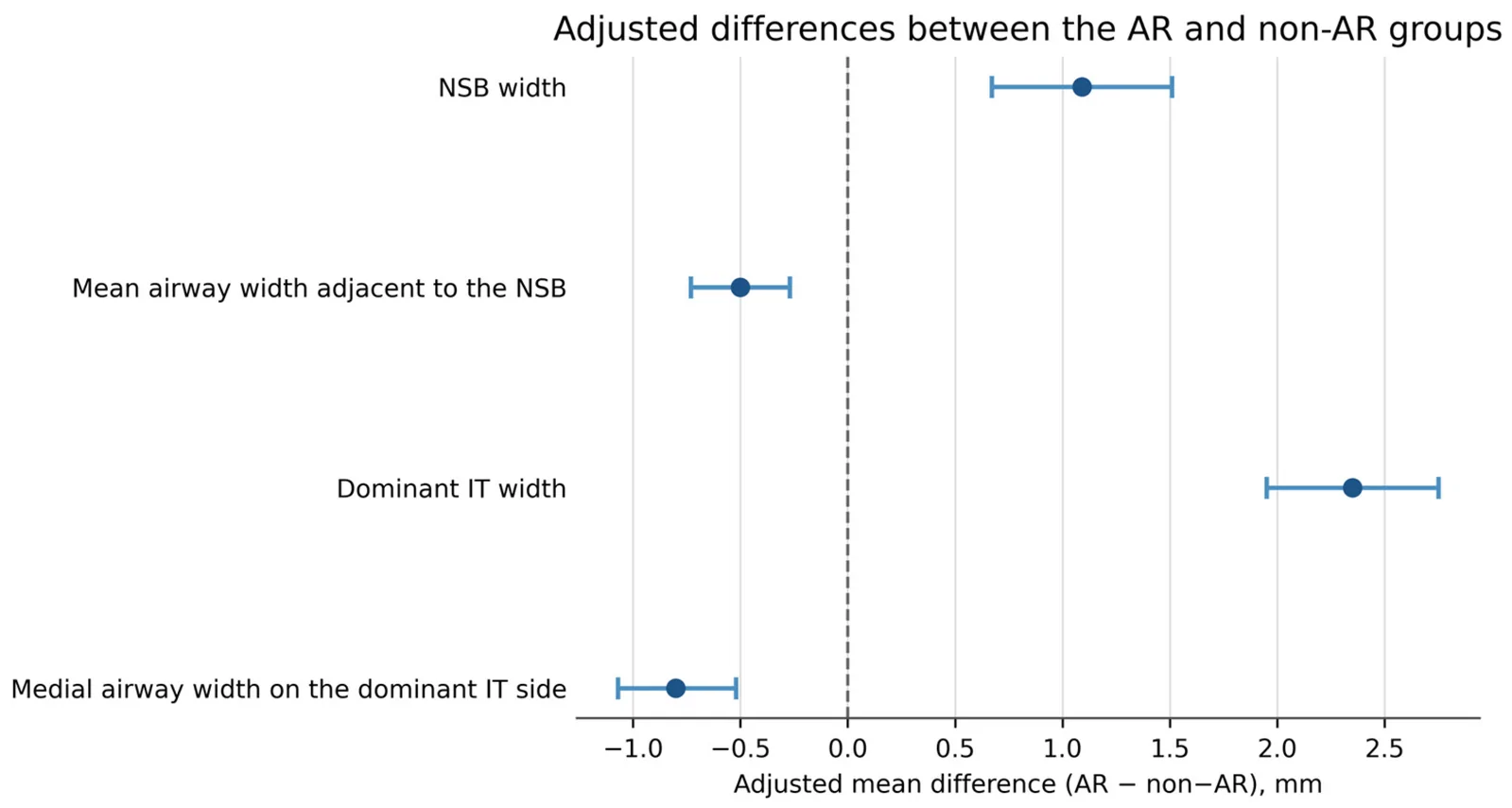
Adjusted differences in the four primary morphometric outcomes between the AR and non-AR groups. Points represent adjusted mean differences (AR − non-AR) from models including age, sex, and septal deviation angle; horizontal error bars represent 95% confidence intervals. Positive values indicate greater measurements in the AR group, whereas negative values indicate smaller measurements in the AR group. AR, allergic rhinitis; NSB, nasal septal body; IT, inferior turbinate.

NSB and dominant inferior turbinate widths were greater in the AR group than in the non-AR group, whereas the mean airway width at the NSB level and the medial airway width on the dominant inferior turbinate side were smaller. All four comparisons remained statistically significant after Holm correction. Adjustment for age, sex, and septal deviation angle did not alter the direction or statistical significance of the associations with AR (Table 2; Figure 2).

Figure 2 visually summarizes the adjusted between-group differences. The AR group showed positive adjusted differences in NSB and dominant IT width and negative adjusted differences in the corresponding airway measures; the largest adjusted between-group difference was observed for dominant IT width.

### 3.4. Correlation Analyses

In the unadjusted analysis, NSB width was positively correlated with dominant inferior turbinate width. This association remained significant in the partial correlation analysis adjusted for age, sex, and septal deviation angle but was no longer significant after AR status was added to the model. Negative correlations were observed between NSB width and mean airway width at the NSB level, as well as between dominant inferior turbinate width and the ipsilateral medial airway width. A positive correlation was also found between the airway dimensions measured at the two anatomical levels. The latter three associations remained significant after false discovery rate correction in the fully adjusted analyses, including AR status (Table 3).

### 3.5. Adjusted Association and Interaction Models

**Figure 3:**
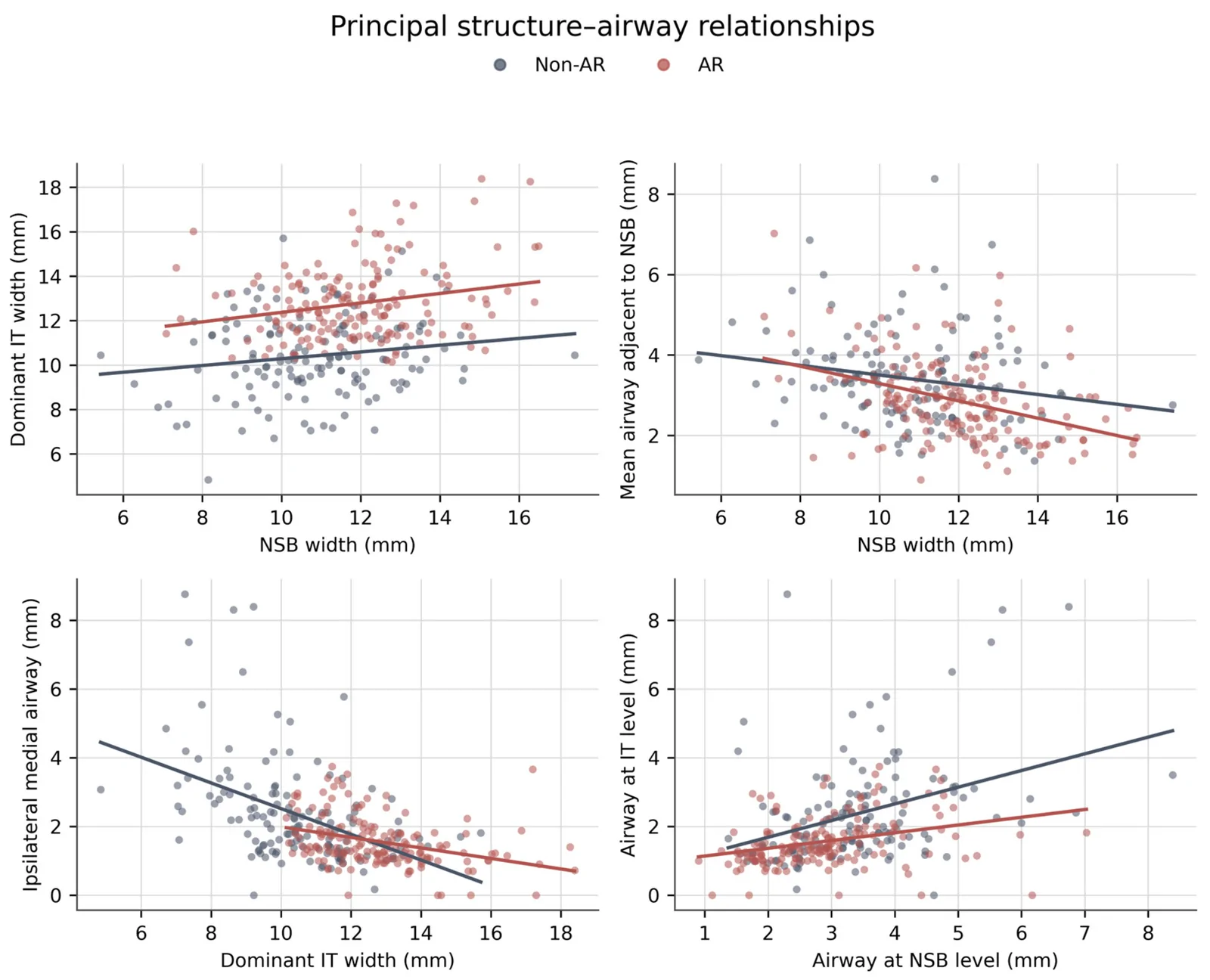
Principal structure–airway relationships by AR status. Upper left: NSB width versus dominant IT width; upper right: NSB width versus mean airway width adjacent to the NSB; lower left: dominant IT width versus ipsilateral medial airway width; lower right: airway width at the NSB level versus airway width at the dominant IT level. Points represent observed measurements, and group-specific unadjusted linear trends are shown for visualization. Formal covariate-adjusted slope and AR-status interaction estimates are reported in Table 4. AR, allergic rhinitis; NSB, nasal septal body; IT, inferior turbinate.

The slope of the association between NSB width and the adjacent airway dimension was negative in both groups; however, the AR-status × NSB-width interaction was not significant after false discovery rate correction. The slope of the association between inferior turbinate width and medial airway width was also negative in both groups, and a significant AR-status × IT-width interaction was observed. Interactions with AR status were not significant for either the NSB-width–IT-width association or the association between airway dimensions measured at the two anatomical levels (Table 4; Figure 3).

Figure 3 displays the four principal structure–airway relationships by AR status. The group-specific fitted trends illustrate the direction and relative magnitude of the associations summarized in Table 3 and Table 4; inferential conclusions regarding differences in slopes by AR status are based on the adjusted interaction models in Table 4 rather than on visual comparison of the fitted lines alone.

### 3.6. Sensitivity Analyses 

Bilateral sensitivity analyses were consistent with the principal structure–airway associations: greater NSB width remained associated with smaller adjacent airway width (B = −0.167, 95% CI −0.234 to −0.100; q < 0.001), greater IT width with smaller ipsilateral medial airway width (B = −0.310, 95% CI −0.362 to −0.258; q < 0.001), and NSB- and IT-level airway dimensions remained positively associated (B = 0.389, 95% CI 0.275 to 0.504; q < 0.001). These models included 602 side-specific observations from 301 patients.

Thirteen airway measurements of 0 mm were recorded across the four side-specific airway variables. Of these, three patients contributed a 0-mm value to the NSB-level outcome and six patients to the dominant IT-level outcome; the remaining zero values occurred on the non-dominant IT side and therefore did not enter the corresponding primary outcome. Excluding the affected patients did not materially change the AR-associated airway differences. The adjusted AR–non-AR difference was −0.504 mm with all observations and −0.546 mm after exclusion (*n* = 298) for the NSB-adjacent airway, and −0.796 versus −0.760 mm (*n* = 295) for the IT-level medial airway (all *p* < 0.001).

## 4. Discussion 

This study evaluated the NSB and IT together with their immediately adjacent airway passages in a single cohort while limiting the influence of marked septal deformity. Three principal findings emerged. First, patients with clinically diagnosed AR had greater total transverse NSB and dominant IT widths and smaller corresponding airway dimensions at the time of CT; the between-group morphometric difference was substantially larger at the IT level. Second, the positive unadjusted relationship between NSB and IT width became weak and non-significant after AR status was added to the model, whereas the inverse relationships between each structure and its adjacent airway persisted. Third, a significant AR-status × IT-width interaction was observed for medial airway width, whereas the corresponding NSB-width interaction was not significant. Taken together, these findings are consistent with a multilevel morphometric pattern associated with AR rather than an isolated difference in a single mucosal structure, while also indicating that the NSB and IT should not be assumed to show identical relationships with the adjacent airway.

The large between-group difference in dominant IT width is consistent with the established involvement of turbinate mucosa in AR-related congestion. Allergic inflammation promotes vasodilatation, vascular permeability, edema, and glandular activity [5,6], and local IgE can be demonstrated within IT tissue [7]. Histological studies also suggest that chronic allergic rhinitis can be accompanied by mucosal remodeling, eosinophilia, mast-cell accumulation, and subepithelial edema [22]. Nevertheless, radiological evidence has been heterogeneous. Al Sharhan et al. did not identify a significant difference in IT dimensions between allergic and non-allergic rhinitis [20], whereas studies of septal deviation have demonstrated substantial associations between local geometry and IT mucosa, bone, and volume [14,15,16]. These discrepancies illustrate why control of septal anatomy and definition of the measured side are important. In the present cohort, restriction of the cohort to septal deviation angles <8° and adjustment for residual septal deviation reduced one major source of anatomical confounding, while use of the numerically dominant IT was intended to capture the side with the greatest local turbinate burden. Importantly, bilateral sensitivity analyses yielded a similar IT–airway association, indicating that the result was not dependent on this side-selection strategy.

The NSB findings extend a developing body of evidence rather than standing alone. Larger NSB dimensions have been reported in patients with sinonasal disease by Gelera et al. [11] and in patients with AR, IT hypertrophy, or other sinonasal pathology by Hizli et al. [17] and Çoban et al. [18], whereas a smaller series found no difference between AR and non-AR groups [19]; the imaging studies relevant to the NSB in AR are summarized in Supplementary Table S2. The group means observed here (11.9 mm in AR and 10.8 mm in non-AR participants) are close to those reported by Gelera et al. [11] for sinonasal disease, predominantly AR, versus controls (12.7 versus 10.5 mm) and by Çoban et al. [18] (12 versus 11 mm), and of the same order as those reported by San et al. [19] (approximately 11 mm in both groups), which supports the comparability of the NSB width measurement across studies. Our results confirm the direction of these earlier observations in a septum-controlled cohort of 301 patients and add several elements that the earlier NSB studies in AR did not provide. Septal geometry was controlled by restricting the cohort to deviation angles below 8° and by adjusting for the residual angle, whereas the earlier NSB studies in AR excluded only severe deviation, summed the two sides, included deviated septa, or did not address it. The NSB and the dominant IT were quantified in the same patients together with the air column adjacent to each structure, an approach that Hirai et al. [21] applied to septal deviation and that is applied here to the comparison of AR and non-AR patients; their demonstration that all four widths depend on septal geometry also supports the restriction and adjustment used in the present cohort. The NSB–IT association was re-estimated after adjustment for AR status, which showed that the crude correlation is largely accounted for by AR status, and AR-status × structure interactions were tested. Finally, intraobserver and interobserver reliability, limits of agreement, and MDC95 were reported for every measurement, which none of the studies summarized in Supplementary Table S2 reported. This integrated design permits the septal and turbinate components of the nasal airway to be considered within a common geometric framework, and the effect sizes obtained in the same cohort (*g* = 0.61 for NSB width versus 1.35 for IT width) quantify how much more strongly AR is associated with the IT than with the NSB.

The attenuation of the NSB–IT correlation after adjustment for AR is also informative. Serial imaging has demonstrated that physiological changes in NSB size correlate with changes in IT size during the nasal cycle [23], supporting some degree of coordinated dynamic behavior. In our cross-sectional data, the NSB–IT association was markedly attenuated after AR status was included in the model, indicating that the crude correlation contained a substantial between-group component. One possible explanation is that both measurements are associated with the allergic mucosal environment; however, the present data cannot distinguish this from nasal-cycle effects or other between-group influences. The data also cannot determine how much of the remaining variation reflects persistent remodeling, residual medication effects, environmental conditions, or stable individual anatomy. Longitudinal imaging under standardized decongestion, allergen-exposure, or medical-treatment conditions would be required to separate these components and to determine whether the NSB responds to anti-allergic treatment in the same way as the inferior turbinate.

The structure–airway analyses provide the most direct anatomical extension beyond prior NSB-size studies. Greater NSB width remained associated with a smaller airway immediately lateral to the NSB after adjustment for AR and other covariates, and greater IT width remained associated with a smaller ipsilateral medial airway. These associations were also present in bilateral sensitivity analyses. The finding is geometrically intuitive and anatomically relevant because greater width of each mucosal structure was associated with a smaller local air column at the same anatomical level rather than representing an isolated soft-tissue measurement. A recent CT case–control study found that septal spur and vomer deviation, rather than SSB deviation, more clearly distinguished patients with clinically relevant nasal obstruction from controls, underscoring that the obstructive relevance of septal anatomy is region-specific [30]. At the same time, smaller airway dimensions on CT should not be equated with airflow resistance or symptom severity. CT depicts geometry at one moment, whereas rhinomanometry measures pressure–flow relationships and acoustic rhinometry estimates cavity geometry using reflected sound; patient-reported obstruction adds a further perceptual dimension [24,25,26,27,28]. This distinction is particularly important in AR, in which mucosal congestion can fluctuate rapidly.

The significant AR-status × IT-width interaction deserves a cautious interpretation. The inverse relationship between IT width and medial airway width was present in both groups but was less steep in the AR group. One possibility is that, within the greater range of IT widths observed in the AR group, additional differences in measured width correspond to proportionally smaller differences in the remaining medial air column because other aspects of nasal geometry may become limiting. Alternatively, differences in mucosal compressibility, side-to-side nasal-cycle phase, or the distribution of hypertrophy within the turbinate may alter the linear relationship between a single width measurement and airway caliber. Because this interaction was a secondary analysis derived from static two-dimensional measurements, it should be regarded as hypothesis-generating rather than as evidence of a distinct AR-specific biomechanical mechanism.

Measurement reproducibility is a notable strength of the study. The CT datasets were reoriented before measurement, anatomical landmarks were standardized in a consensus session, observers were blinded to clinical group, and both intraobserver and interobserver reliability were high. These steps were intended to reduce variability related to image orientation, section selection, and landmark identification. Recent CT-based sinonasal morphometric work in *Diagnostics* has similarly used standardized multidimensional measurements and interobserver ICCs to document reproducible anatomical assessment [29]. In our study, the lower bounds of all ICC confidence intervals exceeded 0.75, and dominant-side classification also showed high kappa agreement. This supports the technical robustness of the measurements, although reproducibility does not by itself establish clinical validity.

Comparison of the between-group differences with the minimum detectable change (MDC95) values in Supplementary Table S1 helps to define the level at which the findings apply. MDC95 is a bound on measurement precision—the smallest difference between two measurements in the same individual that exceeds measurement error with 95% confidence—and not a criterion of clinical significance. The MDC95 values were estimated for the side-specific measurements; MDC95 was not computed directly for the derived patient-level outcomes—the bilateral mean airway width adjacent to the NSB and the dominant-side IT and airway widths, whose definition also involves the selection of the dominant side—so the side-specific values provide context on measurement uncertainty rather than exact benchmarks for these outcomes. Within this limitation, the adjusted between-group differences for total transverse NSB width (1.09 mm) and for the two airway outcomes (−0.50 and −0.80 mm) were smaller than the side-specific MDC95 values (approximately 1.4–2.1 mm), and the difference in dominant IT width (2.35 mm) was of the same order as the side-specific MDC95 values for IT width (2.0–2.5 mm). The AR-associated pattern is therefore well supported at the group level, where measurement error averages out across 301 patients, whereas in an individual patient a single measurement is subject to error of a magnitude comparable to the group differences themselves, and the effect sizes (*g* = 0.61 and 1.35) imply considerable overlap of individual values between the groups. The primary reason that the present data do not provide thresholds for patient-level use is, however, that no threshold was derived or validated against symptoms or objective nasal function; the measurement-uncertainty considerations above indicate that any such validation would also need to take measurement error into account.

From a clinical–anatomical perspective, the findings indicate that in patients with AR the medial septal compartment and the lateral turbinate compartment both differ from non-AR anatomy at the time of CT, and that each is accompanied by a narrower air column at its own level. For the clinician evaluating AR-related nasal obstruction, this supports extending the structural assessment beyond the inferior turbinate: when coronal CT is available, the NSB and the airway immediately lateral to it can be inspected on the same coronal images already obtained for the IT, and a prominent NSB adjacent to a narrow upper air column may indicate a second, septal site of potential obstruction near the internal nasal valve. This is relevant because both compartments are amenable to treatment: inferior turbinate reduction remains effective in appropriately selected patients, although outcomes vary according to technique, patient characteristics, and the underlying cause of obstruction [31,32], and the NSB is an emerging therapeutic target with growing supporting evidence. Temperature-controlled radiofrequency reduction of the septal swell body has produced sustained improvement in nasal obstruction symptoms in selected symptomatic patients [33,34], and a recent systematic review and meta-analysis reported improvement in both patient-reported obstruction scores and objective airway measures after septal swell body reduction [35]. The present study does not identify which patients benefit from these procedures—that requires symptom scores, objective nasal function testing, and treatment outcomes—but it provides the quantitative anatomical basis for such studies by demonstrating that in AR the NSB and IT should be assessed together with their adjacent air columns rather than as isolated structures. Within this framework, CT remains one component of a multimodal evaluation that integrates history, endoscopy, validated symptom measures, and selective objective testing when clinically indicated [24,25,36]. Surgical treatment of rhinitis can improve symptoms in appropriately selected patients [37]; the morphometric differences reported here, however, are group-level anatomical observations rather than diagnostic criteria for AR or indications for intervention.

The AR classification also warrants specific consideration. The diagnosis was based on compatible history, symptoms, and examination/endoscopic findings, with skin-prick or serum-specific IgE testing performed when clinically indicated rather than uniformly. This reflects routine clinical practice but introduces diagnostic heterogeneity. Current consensus documents emphasize that AR diagnosis depends on the clinical context and evidence of allergen sensitization when confirmation is required [1,4]. Moreover, local allergic mechanisms can be present in nasal tissue even when systemic markers are absent; turbinate-specific IgE studies illustrate the complexity of distinguishing allergic, non-allergic, and local allergic phenotypes [7]. The non-AR group in this study should therefore be interpreted as a clinically defined comparison group rather than a uniformly proven nonatopic population, and unrecognized atopy or local allergic rhinitis could not be excluded in this group. If such misclassification was nondifferential with respect to the CT measurements, it would be expected to bias the between-group differences toward the null, so that the reported effects may underestimate the true differences; however, the direction and magnitude of any resulting bias cannot be determined from the available data.

Several limitations related to the measurements should be acknowledged. Total transverse NSB width was recorded as a single transverse dimension that necessarily included the intervening cartilaginous septum, because cartilage and mucosa cannot be separated on CT; the between-group difference therefore reflects the combined mucosal–cartilaginous dimension rather than mucosal thickness alone, although there is no established mechanism by which septal cartilage thickness would differ systematically with allergic rhinitis. Furthermore, no histological confirmation was available, and an increased soft-tissue width on CT cannot distinguish transient mucosal edema or vascular engorgement from glandular or stromal remodeling; the morphometric differences therefore describe the state of the tissue at the time of CT and do not indicate their underlying histological basis. Because the coronal section was selected on the basis of the dominant inferior turbinate, the contralateral measurements were obtained on a section optimized for the opposite side; the bilateral patient-clustered models are therefore supportive rather than confirmatory, and direct right–left comparisons were not undertaken. The distribution of the dominant inferior turbinate side also differed between the groups in an uncorrected secondary comparison. Because the dominant side was assigned from the numerical width measurements rather than from a fixed anatomical side, this imbalance determines which side contributed to the primary outcomes rather than the magnitude of the measurements themselves, and the bilateral models that included both sides showed the same associations. The analysis was based on two-dimensional coronal measurements rather than volumetric segmentation or computational airflow modeling. Although the cohort was restricted to septal deviation angles < 8° and the analyses were adjusted for residual septal deviation, subtle three-dimensional septal geometry may still influence regional airway dimensions. In addition, because each structure and its adjacent air column were measured within the same local space on the same coronal section, the inverse structure–airway associations partly reflect a geometric dependency between opposing surfaces and are reported as morphometric relationships rather than as evidence of a biological mechanism.

The remaining limitations relate to the study design. The retrospective cross-sectional design precludes causal inference and captures only one phase of the nasal cycle. Although, under departmental practice, CT was obtained before medical treatment for rhinitis and was not performed in patients under active treatment, the interval between discontinuation of any previous rhinitis medication and imaging could not be verified retrospectively; residual medication effects therefore cannot be excluded. Recent allergen exposure and allergic disease activity likewise could not be standardized, and each may have altered mucosal volume at the time of imaging. Symptom status at the time of CT, the severity of AR according to the Allergic Rhinitis and its Impact on Asthma (ARIA) classification, and the duration of rhinitis symptoms could not be determined from the available retrospective records, and the absence of validated obstruction scores, rhinomanometry, acoustic rhinometry, or peak nasal inspiratory flow prevents direct linkage of morphology to symptoms or physiological resistance [24,25,26,27,28,38]. For the same reasons, and in view of the MDC95 considerations discussed above, the present study does not provide a validated, clinically meaningful threshold for total transverse NSB width, IT width, or adjacent airway dimensions for use in individual patients. No validated cut-off has been established in the literature either: the criteria proposed so far, such as the classification of an NSB wider than 1.5 cm as markedly enlarged [18], are descriptive and have not been validated against symptoms or nasal function. Furthermore, the cross-sectional design cannot determine whether the observed morphometric differences are reversible, persist over time, or respond to medical treatment of AR; this requires longitudinal assessment in relation to disease activity and treatment. CT examinations were obtained for clinical reasons, creating the possibility of referral and selection bias. Finally, the study was performed at a single center, and the findings require external validation in prospectively characterized populations with standardized allergy testing, documented AR severity and medication status, symptom assessment, and, ideally, repeated imaging before and after treatment.

The study also has several strengths. It included a relatively large cohort for a focused CT morphometry investigation, used a predefined set of primary outcomes with multiplicity correction, reported effect sizes and confidence intervals, and showed that the principal between-group differences remained after adjustment for age, sex, and septal deviation angle. Both intraobserver and interobserver reproducibility were quantified, the dominant-side approach was challenged by bilateral sensitivity analyses, and zero-contact airway measurements were examined separately. Most importantly, the study evaluated opposing mucosal structures and their local airways within the same analytical framework. This permits a more anatomically integrated interpretation of AR-associated nasal anatomy than studies that evaluate the NSB or IT in isolation.

## 5. Conclusions

In conclusion, at the time of CT, patients with AR had greater total transverse NSB and IT widths and smaller immediately adjacent airway dimensions, with a substantially larger between-group morphometric difference at the IT level. The attenuation of the NSB–IT association after adjustment for AR suggests that part of the observed relationship may reflect differences between the AR and non-AR groups, whereas the persistent inverse structure–airway associations are consistent with local geometric relationships between these structures and the adjacent air column. Overall, the findings are consistent with a multilevel morphometric framework for describing AR-associated nasal anatomy rather than an isolated turbinate-based description. Because CT provides anatomical rather than functional information, these measurements should not be interpreted as diagnostic markers of AR or as indications for NSB- or IT-directed treatment. Prospective studies integrating standardized allergy characterization, symptom assessment, objective airflow testing, and longitudinal or volumetric imaging, including imaging before and after treatment, are needed to determine their clinical significance.

## Figures and Tables

**Table 1:** Demographic and baseline characteristics of the AR and non-AR groups.

Characteristic	AR (*n* = 163)	Non-AR (*n* = 138)	Test	Effect (95% CI)	*p*
Age, years	48.20 ± 14.87	48.74 ± 15.26	Welch *t*	−0.54 (−3.98, 2.89)	0.755
Septal deviation angle (°)	5.15 ± 1.50	5.02 ± 1.63	Welch *t*	0.13 (−0.23, 0.49)	0.468
Female, *n* (%)	83 (50.9)	74 (53.6)	Chi-square	-	0.640
Right dominant side, *n* (%)	83 (50.9)	86 (62.3)	Chi-square	-	0.047

Continuous variables are presented as mean ± standard deviation, and categorical variables as *n* (%). For continuous variables, the effect estimate represents the mean difference between the AR and non-AR groups (AR − non-AR). For sex and dominant side, only one category is presented; the complementary category corresponds to the remaining patients. AR, allergic rhinitis; CI, confidence interval.

**Table 2:** Comparison of the primary morphometric outcomes between the AR and non-AR groups.

Outcome	AR	Non-AR	Unadjusted Difference (95% CI)	Effect Size	Holm *p*	Adjusted AR − Non-AR Difference (95% CI)
Total transverse NSB width	11.92 ± 1.81	10.80 ± 1.89	1.12 (0.70, 1.54)	*g* = 0.605	<0.001	1.09 (0.67, 1.51)
Mean airway width adjacent to the NSB	2.81 [2.08–3.42]	3.32 [2.56–3.92]	−0.52 (−0.75, −0.31)	*r*_rb_ = −0.295	<0.001	−0.50 (−0.73, −0.27)
Dominant IT width	12.78 ± 1.69	10.41 ± 1.83	2.37 (1.97, 2.78)	*g* = 1.350	<0.001	2.35 (1.95, 2.75)
Medial airway width on the dominant IT side	1.40 [1.08–1.85]	2.01 [1.38–2.90]	−0.52 (−0.76, −0.33)	*r*_rb_ = −0.363	<0.001	−0.80 (−1.07, −0.52)

All measurements are given in millimeters. Width measurements are presented as mean ± standard deviation and were compared using Welch’s *t* test, whereas airway measurements are presented as median [interquartile range] and were compared using the Mann–Whitney *U* test. The unadjusted difference is the mean difference (AR − non-AR) for width measurements and the Hodges–Lehmann location shift (HL) for airway measurements; the effect size is Hedges’ *g* for width measurements and the rank-biserial correlation coefficient (*r*_rb_) for airway measurements. Holm *p* values refer to the four primary unadjusted between-group comparisons; the adjusted models included age, sex, and septal deviation angle and used HC3 heteroscedasticity-robust standard errors, and all adjusted differences remained significant at *p* < 0.001. HL, Hodges–Lehmann location shift; *r*_rb_, rank-biserial correlation coefficient; NSB, nasal septal body; IT, inferior turbinate; AR, allergic rhinitis; CI, confidence interval.

**Table 3:** Spearman and partial Spearman correlations between anatomical and airway measurements.

Association	Unadjusted rho (95% CI)	Partial rho Without AR	Partial rho Including AR	FDR q
NSB width–dominant IT width	0.257 (0.148, 0.360)	0.238 (0.128, 0.342)	0.089 (−0.025, 0.201)	0.124
NSB width–mean airway width adjacent to the NSB	−0.352 (−0.447, −0.248)	−0.372 (−0.465, −0.269)	−0.319 (−0.418, −0.213)	<0.001
Dominant IT width–ipsilateral medial airway width	−0.509 (−0.588, −0.420)	−0.507 (−0.587, −0.418)	−0.416 (−0.506, −0.318)	<0.001
Mean airway width at the NSB level–medial airway width at the dominant IT level	0.445 (0.350, 0.532)	0.405 (0.305, 0.495)	0.349 (0.245, 0.446)	<0.001

The partial analysis without AR was adjusted for age, sex, and septal deviation angle, whereas the analysis including AR was additionally adjusted for AR status. The reported q values correspond to the fully adjusted family that included AR status; in the unadjusted and AR-free partial families, all four associations remained significant after false discovery rate correction (all q < 0.001). FDR, false discovery rate; NSB, nasal septal body; IT, inferior turbinate; AR, allergic rhinitis; CI, confidence interval; rho, Spearman correlation coefficient.

**Table 4:** Adjusted association and AR-status interaction models.

Model (Predictor → Outcome)	Non-AR Slope B (95% CI)	AR Slope B (95% CI)	AR × Predictor B (95% CI)	Interaction q	Adjusted R^2^
NSB width → IT width	0.134 (−0.020, 0.288)	0.186 (0.013, 0.358)	0.052 (−0.186, 0.289)	0.668	0.342
NSB width → NSB-adjacent airway width	−0.103 (−0.189, −0.018)	−0.227 (−0.326, −0.127)	−0.123 (−0.252, 0.005)	0.121	0.267
IT width → medial airway width	−0.367 (−0.504, −0.230)	−0.150 (−0.228, −0.071)	0.217 (0.059, 0.376)	0.030	0.286
NSB-level airway width → IT-level airway width	0.464 (0.145, 0.782)	0.206 (0.051, 0.361)	−0.258 (−0.607, 0.091)	0.196	0.218

All models were adjusted for age, sex, and septal deviation angle and estimated using HC3 heteroscedasticity-robust standard errors. Model labels are given as predictor → outcome; the predictor is the measurement entered into the AR-status × predictor interaction term, and the slopes are expressed in millimeters per millimeter. Interaction q values were adjusted using the Benjamini–Hochberg procedure. NSB, nasal septal body; IT, inferior turbinate; AR, allergic rhinitis; CI, confidence interval; adjusted R^2^, adjusted coefficient of determination.

## Data Availability

The data that support the findings of this study are available from the corresponding author upon reasonable request. The data are not publicly available due to privacy and ethical restrictions.

## Supplementary Materials

The following supporting information can be downloaded at: https://www.mdpi.com/article/10.3390/diagnostics16213547/s1, Table S1: Intraobserver and interobserver reliability of continuous measurements; Table S2: Imaging studies of the nasal septal body in allergic rhinitis and related conditions compared with the present study. References [11,17,18,19,20,21,23] are cited in the Supplementary Materials.

## Abbreviations

The following abbreviations are used in this manuscript:

AR	Allergic rhinitis
ARIA	Allergic Rhinitis and its Impact on Asthma
CI	Confidence interval
CT	Computed tomography
FDR	False discovery rate
HC3	Heteroscedasticity-consistent (type 3) robust standard error
HL	Hodges–Lehmann location shift
ICC	Intraclass correlation coefficient
IgE	Immunoglobulin E
IT	Inferior turbinate
MDC95	95% minimum detectable change
MRI	Magnetic resonance imaging
NSB	Nasal septal body
SSB	Septal swell body
