# Supplementary material for: Quantitative CT Analysis of Nasal Septal Body and Inferior Turbinate Morphology in Allergic Rhinitis: Structural–Airway Relationships
Source: Diagnostics (Basel). 2026 Oct 28;16(21):3547. doi: 10.3390/diagnostics16213547 (PMC13761893; doi:10.3390/diagnostics16213547)
Supplement: Supplementary file 1 [file diagnostics-16-03547-s001.zip › diagnostics-4584039-supplementary.pdf]

diagnostics

MDPI

Supplementary Materials

Quantitative CT Analysis of Nasal Septal Body and Inferior Turbinate Morphology in Allergic Rhinitis: Structural–Airway Relationships

İbrahim Hıra [1,2] and Hilal Melis Altıntaş [3,*]

[1] Private Otorhinolaryngology Clinic, Ankara, Türkiye; dr.ibrahimhira@gmail.com
[2] Department of Audiology, Faculty of Health Sciences, Ankara Medipol University, Ankara, Türkiye
[3] Department of Anatomy, Faculty of Medicine, Ankara Medipol University, Ankara, Türkiye; hilal.altintas@ankaramedipol.edu.tr
* Correspondence: hilal.altintas@ankaramedipol.edu.tr; Tel.: +90-501-186-98-39

Supplementary Table S1

Supplementary Table S1 reports the detailed intraobserver and interobserver reliability results for each continuous morphometric measurement, including ICCs, mean bias, 95% limits of agreement, and MDC95 values. Dominant-side classification agreement is summarized in the table note.

Table S1. Intraobserver and interobserver reliability of continuous measurements.

Parameter	Intraobserver ICC (95% CI)	Interobserver ICC (95% CI)	Intraobserver bias (95% CI) [limits of agreement]; MDC95	Interobserver bias (95% CI) [limits of agreement]; MDC95
Total transverse NSB width (mm)	0.960 (0.933, 0.976)	0.940 (0.894, 0.965)	0.04 (−0.18, 0.25) [−1.44, 1.52]; 1.47	0.10 (−0.17, 0.37) [−1.73, 1.93]; 1.82
Right airway adjacent to the NSB (mm)	0.940 (0.892, 0.966)	0.920 (0.874, 0.948)	0.03 (−0.18, 0.24) [−1.43, 1.48]; 1.44	−0.04 (−0.31, 0.23) [−1.90, 1.82]; 1.84
Left airway adjacent to the NSB (mm)	0.940 (0.901, 0.965)	0.920 (0.865, 0.949)	−0.01 (−0.24, 0.21) [−1.55, 1.52]; 1.52	0.06 (−0.20, 0.32) [−1.73, 1.86]; 1.78
Right IT width (mm)	0.950 (0.922, 0.967)	0.930 (0.895, 0.952)	0.05 (−0.25, 0.35) [−1.99, 2.09]; 2.02	0.12 (−0.25, 0.49) [−2.43, 2.67]; 2.54
Left IT width (mm)	0.950 (0.919, 0.970)	0.930 (0.888, 0.954)	−0.05 (−0.35, 0.25) [−2.10, 2.00]; 2.03	−0.12 (−0.48, 0.24) [−2.60, 2.36]; 2.47
Right IT medial airway width (mm)	0.930 (0.872, 0.955)	0.910 (0.822, 0.948)	0.05 (−0.22, 0.32) [−1.83, 1.93]; 1.86	−0.02 (−0.32, 0.28) [−2.11, 2.07]; 2.07
Left IT medial airway width (mm)	0.930 (0.841, 0.963)	0.910 (0.802, 0.952)	0.08 (−0.17, 0.33) [−1.63, 1.79]; 1.70	0.10 (−0.19, 0.38) [−1.87, 2.06]; 1.96
Septal deviation angle (°)	0.920 (0.864, 0.949)	0.890 (0.801, 0.938)	−0.13 (−0.34, 0.09) [−1.60, 1.34]; 1.48	−0.20 (−0.44, 0.04) [−1.86, 1.46]; 1.68

 https://doi.org/10.3390/diagnostics16213547

Reliability was assessed in a randomly selected subset of 50 patients with genuine repeated measurements (n=50). Single-measure absolute-agreement intraclass correlation coefficients were estimated using a two-way mixed-effects model for intraobserver reliability and a two-way random-effects model for interobserver reliability, with bootstrap 95% confidence intervals from 5000 patient-level resamples. Limits of agreement were calculated as the mean bias ± 1.96 times the standard deviation of the paired differences; the standard error of measurement (SEM) was calculated as SD × √(1 − ICC) and MDC95 as 1.96 × √2 × SEM. The bootstrap confidence interval for the mean bias included zero for every parameter, providing no evidence of systematic measurement shift. For dominant-side classification, intraobserver agreement was 96%, κ=0.918 (95% CI, 0.791-1.000), and interobserver agreement was 92%, κ=0.838 (95% CI, 0.673-0.960). Bias, limits of agreement, and MDC95 are expressed in millimeters for linear measurements and degrees for the septal deviation angle. ICC, intraclass correlation coefficient; CI, confidence interval; MDC95, 95% minimum detectable change.

Supplementary Table S2

Supplementary Table S2 summarizes the published imaging studies that have addressed the nasal septal body (NSB) in allergic rhinitis (AR) and related conditions, alongside the present study, with respect to population and medication status, AR definition, measurements, handling of septal deviation, reliability assessment, and principal NSB finding; the CT comparison of inferior turbinate dimensions between AR and non-AR patients by Al Sharhan et al. [20], which did not measure the NSB, and the CT study of septal deviation by Hirai et al. [21], which measured the NSB and both adjacent air columns but did not compare AR and non-AR patients, are included for completeness. Study names are linked to the corresponding articles through their DOIs.

Table S2. Imaging studies of the nasal septal body in allergic rhinitis and related conditions compared with the present study.

Study	Population (n) and design	AR definition	NSB measurement	IT and adjacent airway	Septal deviation	Reliability	Main NSB finding
San et al., 2014 [19]	AR 40 / non-AR 30; retrospective CT; patients using nasal sprays, oral antihistamines, steroids, or leukotriene antagonists excluded	Clinical diagnosis + total IgE + SPT	Horizontal width at the widest coronal level	Not measured	Not addressed	Not reported	No difference in NSB width (approximately 11 mm in both groups)
Gelera et al., 2017 [11]	Sinonasal disease 312 (AR in 285, alone or with CRS) / controls 93; retrospective CT; patients under prolonged medication included	ARIA criteria + SPT/MAST	Horizontal width at four septal levels	Not measured	Severe deviation excluded; not analyzed	Single reader; not reported	NSB wider at all levels (widest level 12.7 vs. 10.5 mm)
Al Sharhan et al., 2018 [20]	AR 49 / non-AR 41, all with septal deviation requiring septoplasty; CT and acoustic rhinometry after at least 2 weeks without medical treatment	History + MAST + total IgE	Not measured	IT widths and medial mucosa; septum–IT and choanal space	All deviated; compared by side	Not reported	No AR-related difference in IT or airway dimensions

Study	Population (n) and design	AR definition	NSB measurement	IT and adjacent airway	Septal deviation	Reliability	Main NSB finding
Hizli et al., 2020 [17]	ITH with AR 54 / ITH without AR 52 / controls 49; retrospective CT; medication status not stated	Symptoms + SPT	Area (coronal, both sides summed), length and width (axial)	IT area; airway not measured	Sides summed to offset asymmetry	Not reported	NSB area and width greater with AR; NSB area correlated with IT area (rho 0.65–0.67)
Çoban et al., 2020 [18]	AR only 76; AR + sinonasal pathology 211; sinonasal pathology only 201 / controls 89; retrospective CT; patients treated for sinonasal or allergic complaints excluded	ARIA criteria + SPT + IgE	Widest horizontal width (mean of two readings)	Not measured	Counted as a sinonasal pathology (present in 87% of the sinonasal-pathology groups); not adjusted	Single reader (mean of two readings); not reported	NSB wider in all patient groups than in controls (1.2 vs. 1.1 cm); no difference among patient groups
Wong et al., 2020 [23]	34 patients without sinonasal disease; two CT scans 1.25 ± 1.13 years apart; medication status not stated	No AR grouping	Width and height at the widest level	Total IT width; airway not measured	Not addressed	Not reported	Change in NSB width correlated with change in IT width (r = 0.35)
Hirai et al., 2025 [21]	152 patients undergoing endoscopic endonasal surgery (304 sides); retrospective CT; decongestant use before CT excluded	No AR grouping; AR recorded as comorbidity only	Unilateral width, lateral NSB border to bony septum, per side (not comparable with total transverse width)	IT width; airway lateral to the NSB and between the IT and the septum, per side	Graded by septal angle (<9°, 9–15°, >15°); convex and concave sides compared	Not reported	NSB, IT, and both airway widths narrower on the convex side; side difference in NSB width correlated with the septal angle (r = 0.81)
Present study	Clinically diagnosed AR 163 / non-AR 138; retrospective CT; CT obtained before medical treatment under departmental practice, medication history not recorded	Clinical diagnosis; SPT/sIgE when available	Total transverse width at the widest level	Dominant and bilateral IT width; airway adjacent to the NSB and medial to the IT	Restricted to <8° and adjusted for residual angle	Two observers; ICC, limits of agreement, MDC95	NSB g = 0.61 vs. IT g = 1.35; inverse structure–airway associations at both levels; NSB–IT correlation attenuated after AR adjustment; AR × IT interaction

Reference numbers in brackets refer to the reference list of the main manuscript; study names are hyperlinked to the articles via their DOIs. AR, allergic rhinitis; ARIA, Allergic Rhinitis and its Impact on Asthma; CRS, chronic rhinosinusitis; CT, computed tomography; ICC, intraclass correlation coefficient; IgE, immunoglobulin E; IT, inferior turbinate; ITH, inferior turbinate hypertrophy; MAST, multiple allergen simultaneous test; MDC95, 95% minimum detectable change; NSB, nasal septal body; sIgE, serum-specific IgE; SPT, skin-prick test.

Disclaimer/Publisher's Note: The statements, opinions and data contained in all publications are solely those of the individual author(s) and contributor(s) and not of MDPI and/or the editor(s). MDPI and/or the editor(s) disclaim responsibility for any injury to people or property resulting from any ideas, methods, instructions or products referred to in the content.

https://doi.org/10.3390/diagnostics16213547
